# Vertebrated Animals in the Human Stomach

**Published:** 1877-02-20

**Authors:** 


### Editorial and Miscellaneous.

☞ All communications relating to the business of THE RECORD, for the year 1877, must be addressed to
DR. R. C. WORD,
Business Manager, South. Med. Rec.,
Atlanta, Ga.

☞ Brief and practical communications are solicited on all subjects pertaining to medicine, also reports of cases in practice.

☞ Send money by check, postal order, or registered letter.

☞ Write your name, post-office, county and State plainly.

### VERTEBRATED ANIMALS IN THE HUMAN STOMACH.

Dr. Fisher, of Ohio, reports to the *Nashville Journal of Medicine*, a case of a mouse, swallowed by a lady from a pitcher of water, which remained alive in the stomach a period of four months, when it was ejected by vomiting. The editor, while publishing the case, and uttering nothing disparaging to the doctor's veracity, evidently regards him the dupe of a hysterical trick. We will here mention the facts of a case given to us by Dr. Gordon, of LaFayette, Ga., a respectable physician, who affirmed that the facts were positively true as related. We repeat from memory, and will, therefore, not attempt to give the dates, or describe accurately, the symptoms. The doctor stated that he had been somewhat annoyed by frequent calls to visit a negress suffering with paroxysms of pain and cramps in the stomach, described by the patient as a crawling, moving, sensation, and a sense of oppression and choking. These symptoms had recurred at frequent intervals for two or three years, each successive attack seeming to grow worse, until at length they became almost unsupportable. The doctor, supposing it to be a case of hysteria, prescribed a mixture of hartshorn and assafœteda, which always gave relief for the time. As the attack continued to return from day to day, the doctor at length prepared a quantity of the medicine and directed her to use it *pro re nata*. For eight or ten days after the directions last given, he heard nothing of his patient, but was again sent for in great haste and informed that bones and putrid matter were being discharged from the bowels—a fact which had greatly alarmed the patient. Upon examining the matter thus discharged, he found a number of bones and portions of the skin of a vertebrated animal. These he carefully preserved, and directed them to preserve any other portions that might appear. At a subsequent visit, more had been discharged, and the skeleton complete was found. The Doctor gave us a rib, a vertebra and a claw with a portion of the skin of the animal which we subjected to a close examination.—The claw was very nearly an half inch in length. The tibia and femur were about $\frac{3}{4}$ of an inch each, and articulated in a manner indicative of a crawling, or crab like motion. The rib was slightly curved and nearly an inch in length.—Allowing the corresponding rib to be of equal length, the vertebra interposed, and a reasonable space for the cartilaginous, or membranous connection in front, the animal must have been not less than three inches in circumference, or an inch in diameter, and probably five to six inches in length. The skin resembled that of a cat-fish. We make no comment on this case further than to add that there is in that country an amphibi ous creature called water dog, which has great tenacity of life, and which fully answers the description of the animal described. Touching on the facts as related, we believe them correct, that the Doctor was not deceived by the patient, and that he is a man of truth and integrity. W.

